# Supplementary material for: Discrete simulation analysis of COVID-19 and prediction of isolation bed numbers
Source: PeerJ. 2021 Jun 23;9:e11629. doi: 10.7717/peerj.11629 (PMC8234972; doi:10.7717/peerj.11629)
Supplement: Supplemental Information 1 [file peerj-09-11629-s001.docx]

| The gap in the number of isolated beds | Duration of isolation facilities at full capacity |
| --- | --- |
| 0.6 | 82.1 |
| 0.4 | 33.8 |
| 0.2 | 12.9 |
| 0 | 0 |
