# Supplementary figures and images for: Discrete simulation analysis of COVID-19 and prediction of isolation bed numbers
Source: PeerJ. 2021 Jun 23;9:e11629. doi: 10.7717/peerj.11629 (PMC8234972; doi:10.7717/peerj.11629)

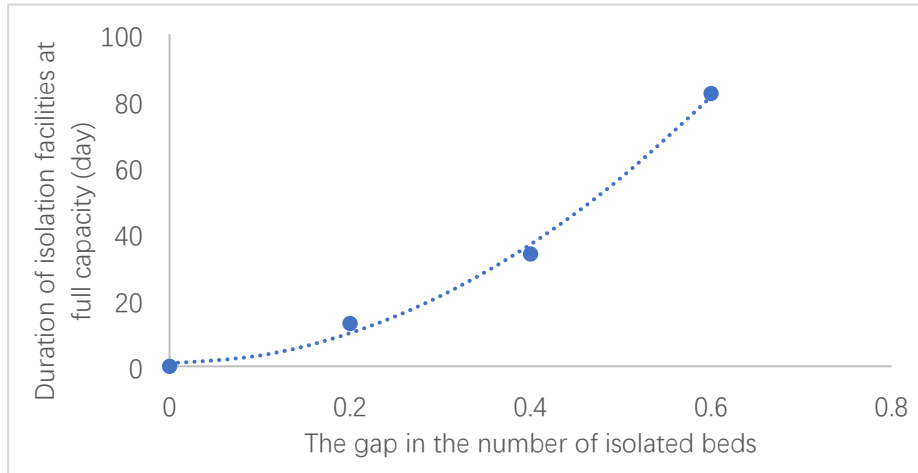

Supplement: Supplemental Information 2 — The corresponding date of the peak inpatient number was delayed with the decrease in isolation capacity. The duration of isolation facilities at their full capacity increased with the lack of isolation beds, which showed a quadratic relationship. [file peerj-09-11629-s002.pdf]
